# Delta Power Is Higher and More Symmetrical in Ischemic Stroke Patients with Cortical Involvement

**DOI:** 10.3389/fnhum.2017.00385

**Published:** 2017-07-28

**Authors:** Chiara Fanciullacci, Federica Bertolucci, Giuseppe Lamola, Alessandro Panarese, Fiorenzo Artoni, Silvestro Micera, Bruno Rossi, Carmelo Chisari

**Affiliations:** ^1^Neurorehabilitation Unit, University Hospital of Pisa University of Pisa, Pisa, Italy; ^2^The BioRobotic Institute, Scuola Superiore Sant’Anna Pisa, Italy; ^3^Translational Neuroengineering Lab, School of Engineering, École Polytechnique Fèdèrale de Lausanne Lausanne, Switzerland

**Keywords:** stroke, cerebrovascular disease, clinical neurophysiology, neuronal plasticity, quantitative electroencephalography, brain asymmetry

## Abstract

A brain injury resulting from unilateral stroke critically alters brain functionality and the complex balance within the cortical activity. Such modifications may critically depend on lesion location and cortical involvement. Indeed, recent findings pointed out the necessity of applying a stratification based on lesion location when investigating inter-hemispheric balance in stroke. Here, we tested whether cortical involvement could imply differences in band-specific activity and brain symmetry in post stroke patients with cortico-subcortical and subcortical strokes. We explored brain activity related to lesion location through EEG power analysis and quantitative Electroencephalography (qEEG) measures. Thirty stroke patients in the subacute phase and 10 neurologically intact age-matched right-handed subjects were enrolled. Stroke patients were equally subdivided in two groups based on lesion location: cortico-subcortical (CS, mean age ± SD: 72.21 ± 10.97 years; time since stroke ± SD: 31.14 ± 11.73 days) and subcortical (S, mean age ± SD: 68.92 ± 10.001 years; time since stroke ± SD: 26.93 ± 13.08 days) group. We assessed patients’ neurological status by means of National Institutes of Health Stroke Scale (NIHSS). High density EEG at rest was recorded and power spectral analysis in Delta (1–4 Hz) and Alpha (8–14 Hz) bands was performed. qEEG metrics as pairwise derived Brain Symmetry Index (pdBSI) and Delta/Alpha Ratio (DAR) were computed and correlated with NIHSS score. S showed a lower Delta power in the Unaffected Hemisphere (UH) compared to Affected Hemisphere (AH; *z* = −1.98, *p* < 0.05) and a higher Alpha power compared to CS (*z* = −2.18, *p* < 0.05). pdBSI was negatively correlated with NIHSS (*R* = −0.59, *p* < 0.05). CS showed a higher value and symmetrical distribution of Delta band activity (*z* = −2.37, *p* < 0.05), confirmed also by a higher DAR value compared to S (*z* = −2.48, *p* < 0.05). Patients with cortico-subcortical and subcortical lesions show different brain symmetry in the subacute phase. Interestingly, in subcortical stroke patient brain activity is related with the clinical function. qEEG measures can be explicative of brain activity related to lesion location and they could allow precise definition of diagnostic-therapeutic algorithms in stroke patients.

## Introduction

Stroke affects 15 million people worldwide every year: it is the second leading cause of death in Europe and one of the main causes of long-term disability (Source: www.escardio.org). After a stroke, brain tissue in the affected vascular territories becomes dysfunctional, and ultimately necrotic. An ischemic lesion affects the functional network architecture of cortical areas in both hemispheres (Murase et al., 2004; Grefkes et al., 2008; Wang et al., 2010; Grefkes and Fink, 2011). A structural lesion resulting from unilateral stroke may critically disturb bilaterally the complex balance of excitatory and inhibitory influences within the cortical network. Clinical features in stroke patients are related to a reduced output from the Affected Hemisphere (AH) and excessive interhemispheric inhibition from the Unaffected Hemisphere (UH) to the AH, as described in a theoretical model known as “interhemispheric competition model” (Kinsbourne, 1980; Murase et al., 2004; Takeuchi et al., 2005; Chisari et al., 2014).

Up to now, cortical activity after a brain injury has been studied by means of neuroimaging approaches including functional magnetic resonance imaging (fMRI) and positron emission tomography (PET). These techniques provided useful insights into the pathophysiological effects of a stroke, as quantitative index of neural network and functional connectivity changes (Westlake et al., 2012). In detail fMRI demonstrated a variability across stroke subjects in brain activation patterns. A previous study described a different activation pattern in patients with cortico-subcortical vs. subcortical lesions despite similar chronic motor impairment, suggesting lesion-specific mechanisms of reorganization (Luft et al., 2004). Following studies showed that motor deficit of subcortical stroke patients during the early period after stroke were associated with pathological interhemispheric interactions among key motor areas (Grefkes et al., 2008). Nevertheless, the low temporal resolution, the necessity of the patients’ collaboration and the high cost contribute to the limited use of these techniques.

A valid alternative method is represented by quantitative Electroencephalography (qEEG): a non-invasive, easy applicable technique characterized by a high temporal resolution but low spatial resolution. qEEG is very sensitive in detecting abnormalities of cerebral rhythms that are typical of stroke. Specific band power activities are considered to be linked to brain functions and in case of stroke, are associated to different degrees of neuronal survival in the ischemic regions and therefore can assume a prognostic value (Niedermeyer, 2005; Kaplan and Rossetti, 2011; Assenza et al., 2013; Finnigan and van Putten, 2013; Rossiter et al., 2014). EEG power is markedly affected in stroke patients with a significant increase in Delta power (1–4 Hz) accompanied by a decrease in Alpha (8–14 Hz) and Beta (14–30 Hz) power producing a diffuse slow-wave EEG pattern (Faught, 1993; Lu et al., 2001). The increasing power of slow rhythms and decreasing power of fast rhythms are directly linked with neuronal metabolism and reflect ischemic injury (Foreman and Claassen, 2012; Wu et al., 2016). In detail a previous study described that Delta and Alpha bands modification are correlated with clinical outcome (Cuspineda et al., 2007). The central role of these frequency bands led to the identification of the quantitative index called Delta/Alpha ratio (DAR), which quantifies the global Delta activity relative to the normative Alpha activity. In literature this index was found to detect delayed ischemeia, in subarachnoid hemeorrhage prior to symptom changes (Claassen et al., 2004; Finnigan and van Putten, 2013). Furthermore several studies showed that an increase in Delta power and/or decrease in Alpha power (greater DAR) was related to poorer outcome in sub-acute stroke patients (Machado et al., 2004; Finnigan et al., 2007; Sheorajpanday et al., 2011) and in chronic acquired brain injury (Leon-Carrion et al., 2009).

Moreover, also the inter-hemispheric balance could be studied with qEEG metrics as the brain symmetry index (BSI), that provides a measure of inter-hemispheric EEG power asymmetry not specific to single frequency bands (van Putten and Tavy, 2004; van Putten, 2007). The BSI is mainly used in the research field for the purposes of stroke prognosis: literature shows that in acute stroke patients a high BSI is positively correlated with a worse neurological status (van Putten and Tavy, 2004; van Putten, 2007) and with a weaker motor recovery 2 months after the acute event (Agius Anastasi et al., 2017).

Although these methods have proved useful to describe the correlation between stroke and clinical status (Simis et al., 2016), to date little is known about brain activity characterization based on stroke location using qEEG. Recent findings using transcranial magnetic stimulation suggest to apply a stratification based on lesion location and to consider patients with cortico-subcortical and subcortical strokes separately (Thickbroom et al., 2015) as already suggested by fMRI findings (Luft et al., 2004). In particular Thickbroom et al. (2015) highlighted that the contralesional motor cortex excitability depends on lesion location and only in subcortical stroke patients the cortical excitability is related to functional status. This result could be meaningful in order to study specific therapeutic approach such as neuromodulation techniques (Thickbroom et al., 2015).

Neurophysiological data in first weeks after the acute event may suffer from wide inter-subject variability. In this regard previous studies highlighted that during the early phase (3 weeks) after stroke the physiological variability is not related to changes in clinical status (Swayne et al., 2008).

In this study, we hypothesized that cortical involvement in stroke could generate differences in brain symmetry and band-specific activity compared to stroke cases without cortical involvement. Therefore, we explored brain electrical activity through qEEG analysis in a cohort of stroke patients recruited within 45 days after the acute event and to establish a characterization based on of lesion location (cortico-subcortical and subcortical lesions). Moreover the purpose was to relate the neurophysiological findings with patients’ neurological status.

## Materials and Methods

A group of 30 ischemic stroke patients (M/F: 19/11; mean age ± SD: 69.5 ± 13.6 years) in the subacute phase of the disease (>10 and <45 days after the stroke) was enrolled at the Neurorehabilitation Unit of University Hospital of Pisa. Inclusion criteria were: (1) age ranged between 18 years and 80 years; (2) first-ever unilateral ischemic stroke; and (3) time from acute event within 45 days. Exclusion criteria were: (1) use of drugs targeting CNS; (2) diagnosis of epilepsy; and (3) MMSE <24. The sample has been divided in two groups according to the lesion site: 15 with cortical-subcortical (CS) and 15 with subcortical (S) lesion. The whole sample was assessed by means of a standard Computed Tomography (CT) scan, performed in the Neuroradiology Department of the University-Hospital of Pisa. Brain CT was performed before the admission at the Neurorehabilitation Unit in a range between 3 days and 10 days after the acute event (Median time = 5 days; Interquartile Range (IQR) = 6 days). Patients were enrolled when the imaging showed stable injuries and indication to not perform further examination was given.

Based on brain CT images, lesions were defined as S (mean age ± SD: 68.92 ± 10.001 years; time since stroke ± SD: 26.93 ± 13.08 days) if they involved the deep white matter inferior to the corpus callosum, including the internal capsule, thalamus, and basal ganglia and spared the cerebral cortex. Otherwise all lesions including also a cortical involvement were defined as CS (mean age ± SD: 72.21 ± 10.97 years; time since stroke ± SD: 31.14 ± 11.73 days). Data about age, gender, side of the lesion (right or left), stroke location, time since stoke at the time of evaluation were obtained from all the subjects (Table 1). Ten neurologically intact age-matched right-handed subjects (M/F: 4/6; mean age ± SD: 62.0 ± 10.3 years) were also included in the study as control group. Clinical and neurophysiological evaluation were performed in a single 2-h-long session.

**Table 1:** Subjects sample details.

Patient	Sex	Age year	Affected hand	Days after stroke	Lesion location	NIH stroke scale
1	Male	67	right	45	Cortico-subcortical	6
2	Male	60	right	45	Cortico-subcortical	6
3	Male	54	left	31	Cortico-subcortical	6
4	Male	65	left	15	Cortico-subcortical	7
5	Female	79	right	23	Cortico-subcortical	4
6	Female	84	left	23	Cortico-subcortical	4
7	Male	85	right	45	Cortico-subcortical	7
8	Male	76	left	26	Cortico-subcortical	3
9	Female	83	right	28	Cortico-subcortical	5
10	Female	80	right	45	Cortico-subcortical	6
11	Female	77	left	14	Cortico-subcortical	4
12	Male	59	left	19	Cortico-subcortical	2
13	Female	73	left	34	Cortico-subcortical	6
14	Female	61	left	14	Cortico-subcortical	2
15	Female	85	left	43	Cortico-subcortical	5
16	Male	71	right	45	Subcortical	5
17	Male	70	right	23	Subcortical	5
18	Male	54	left	22	Subcortical	4
19	Male	59	right	45	Subcortical	7
20	Male	78	right	19	Subcortical	2
21	Male	63	left	19	Subcortical	3
22	Female	74	left	45	Subcortical	3
23	Male	65	right	32	Subcortical	5
24	Male	22	right	21	Subcortical	1
25	Female	82	left	27	Subcortical	3
26	Male	52	right	15	Subcortical	2
27	Male	73	left	11	Subcortical	2
28	Male	80	left	18	Subcortical	8
29	Male	62	left	11	Subcortical	3
30	Female	82	left	45	Subcortical	4

Patients’ neurological status was determined through the National Institutes of Health Stroke Scale (NIHSS). The NIHSS is a tool used by physicians to objectively quantify the impairment caused by the stroke. It is composed of 11 items, each of which scores a specific ability between a 0 and 4. For each item, a score of 0 typically indicates normal function in that specific ability, while a higher score is indicative of some level of impairment. The individual scores from each item are summed in order to calculate patient’s total score (Wityk et al., 1994).

A qEEG recording lasting 10 min with eyes closed was performed. Subjects were seated in a comfortable chair during resting state with eyes closed in an acoustically and electrically shielded room. The EEG and the vertical electrooculogram (EOG) were recorded using a 64-channel DC-coupled monopolar amplifier (Micromed SD MRI, System Plus acquisition software). The montage was in accordance to the 5% 10/20 system (Oostenveld and Praamstra, 2001). After careful scalp preparation, EEG signals were acquired at a sampling rate of 256 Hz by electrodes having contact impedance below 10 kΩ in at least 95% of derivations throughout the experiment (electrode re-gelling was performed whenever required). The reactivity of the EEG to state manipulations was assessed before recording in order to exclude the impact of the state manipulation on the EEG signal. Data containing artifacts due to eye blinks, significant muscle activities and electrode displacement artifacts were removed in an offline visual screening. Although the influence of ocular artifacts with eyes closed is lower than with eyes open, small ocular movements may still be present. Given their stereotyped nature, independent component analysis (ICA) enabled us to identify and remove such residual ocular activity. The EEG signals were offline re-referenced to the two electrodes nearest to the mastoids (Tp7 and Tp8) to obtain monopolar recordings (Menicucci et al., 2014), then high pass filtered with a zero-phase Chebyshev type-2 filter (1 Hz stopband, 2 Hz passband, 80 db attenuation) and low pass filtered with a Chebyshev type 2 filter (45 Hz passband, 48 Hz stopband, 80 dB attenuation). Abnormal data with extreme magnitude (which include mean deviations, jumps and large oscillations) from the continuous dataset were removed by using a customized version of the routine flt_clean_windows (BCIlab) to compute a moving windowed signal power. EEG windowed segments (1 s) were removed if their power exceeded the 90% distribution quantile. Synchronous sudden increases in signal amplitude were detected by computing the difference between the superior and inferior envelopes (shape-preserving piecewise cubic interpolation; Brodlie and Butt, 1991), EEG portions producing values greater than 2.5 standard deviations (in amplitude distribution) were removed. The process followed three consecutive steps: (i) the computation of inferior and superior all-channels envelopes; (ii) the local range setting as the difference between the sum of the superior and inferior all-channels envelopes respectively; and (iii) the detection of movement artifact relating to a suitable threshold on the local range (2.5 standard deviations in amplitude distribution; Artoni et al., 2012b). Bad channels were identified by computing global channel measures (e.g., Kurtosis), and by visual inspection. Eye blinks were identified by computing an adaptive threshold on the moving-windows cross-correlation between the EOG and the frontal EEG channels (Menicucci et al., 2014; Sebastiani et al., 2015; Genna et al., 2017). Finally ICA was applied on the cleaned dataset and the ocular component was removed (Artoni et al., 2012a, 2014; Oddo et al., 2016).

Power spectral density was computed for each channel by averaging periodograms of windowed signal sections (pwelch function in Matlab). The window length was 2 s (512 time points), without zero padding or overlap. On average 8–9 min of artifact-free EEG data per patient were available for power analyses.

The power spectral density was computed for the UH and for the AH. An “average scalp power spectrum” was defined as the mean power spectrum density (PSD) across all scalp electrodes. From the average scalp power spectra, we computed the average PSD across the following frequency bands: Delta (1–4 Hz) and Alpha (8.1–14 Hz). The “area’s power spectrum” was defined as the mean PSD over adjacent electrodes within the following areas: Frontal Area (left side: Fp1, AF3, AF7, F1, F3, F5, F7, right side: Fp2, AF4, AF8, F2, F4, F6, F8), Central Area (left side: FC1, FC3, FC5, FT7, C1, C3, C5, T3, CP1, CP3, CP5; right side: FC2, FC4, FC6, FT8, C2, C4, C6, T4, CP2, CP4, CP6) and Posterior Area (left side: P1, P3, P5, T5, PO7, P03, O1; right side: P2, P4, P6, T6, PO8, P04, O2).

The resulting average power values in the two bands were then used to compute the following qEEG metrics:

1. DAR, i.e., the ratio of mean scalp Delta to Alpha power. We chose to compute this ratio rather than its inverse which has been reported by others (Claassen et al., 2004) because for the DAR a higher score indicates a greater degree of EEG shift towards low-frequency activity (Finnigan et al., 2007).
2. Pairwise derived Brain Symmetry Index (pdBSI). The index evaluates the asymmetry across homologous channel pairs considering the 1–40 Hz range averaged over frequency and number of channel pairs. We used 23 channel pairs: Fp1-Fp2; F7-F8; F3-F4; C3-C4; T3-T4; T5-T6; P3-P4; O1-O2; FC1-FC2; P1-CP2; PO3-PO4; FC5-FC6; CP5-CP6; AF3-AF4; F1-F2; F5-F6; FC3- FC4; FT7-FT8; C1-C2; C5-C6; CP3-CP4; P1-P2; P5-P6. The pdBSI is a numerical value, ranging from zero (perfect symmetry for all channels) to one (maximal asymmetry; Sheorajpanday et al., 2009). We used the pdBSI following previous studies (Sheorajpanday et al., 2009, 2011).
  (1) $$\text{BSI }=\;\sum_{j=1}^{M}\sum_{i=1}^{N}\left|\frac{{\text{AH}}_{ij}-{\text{UH}}_{ij}}{{\text{AH}}_{ij}+{\text{UH}}_{ij}}\right|$$
  with AH_ij_ and UH being the power spectral densities of the signals obtained from pairs of homologous channels (with *i* = 1, 2,…, M) from affected and UH, respectively, at frequency *j* (with *j* = 1, 2,…, N).

Outliers were identified by means of the Grubbs test and they were removed from the sample. As the Shapiro-wilk test showed non-Gaussian distribution of data samples, non-parametric statistics were used.

Mann-Whitney test was used to identify inter-group differences in EEG and Clinical variables. Wilcoxon signed-rank test was performed for the intra-group inter-hemispheric comparison between unaffected and AH in CS and S, and for the comparison between right and left hemisphere in healthy subjects.

Healthy subjects and stroke patients subgroups were compared for age parameters in order to confirm the age comparability between groups.

The time after the acute event of CS and S patients was compared in order to exclude differences in post stroke evolution between the two groups.

The PSD across all scalp was compared between each group and healthy subjects. Inter-group (CS vs. S) and intra-group comparison (AH vs. UH) was performed for PSD frequency bands and DAR over the whole scalp, single hemisphere and frontal, central and posterior area.

Inter-group comparison (CS vs. S) was performed for pdBSI both for the whole scalp and frontal, central and posterior area.

Spearman’s rank correlation coefficient was used to compute correlations between time since stroke and EEG parameters (Delta and Alpha bands, pdBSI and DAR) to evaluate the day-to-day variation in EEG activity.

Moreover Spearman’s rank correlation analysis was performed between qEEG metrics (DAR and pdBSI) and NIHSS score.

Signal processing and analyses were performed offline using MatLab (Mathworks, Natick, MA, USA) with custom scripts based on the EEGLAB toolbox (Delorme and Makeig, 2004). Statistical analysis was performed with SPSS 20.0 software (SPSS Inc. Chicago, IL, USA). Significance of statistical tests was set at *p* < 0.05. Bonferroni correction was applied.

Each patient and healthy subject recruited gave their written informed consent in accordance with the Declaration of Helsinki. This study was authorized by local Ethics committee of Area Vasta Nord Ovest (CEAVNO) for Clinical experimentation, Tuscany (Italy), protocol n° 901.

## Results

CS, S and Healthy subjects did not show significant different in age range. Stroke patients were subdivided according to the ischemic injury localization. No significant difference was found in the timing since stroke between the two groups.

### Clinical Status

**Figure 1:**
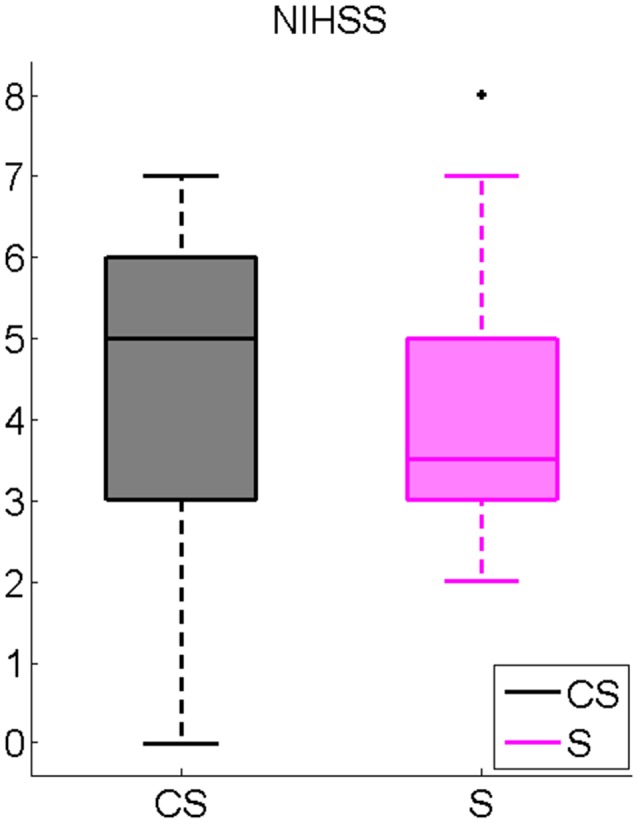
National Institutes of Health Stroke Scale (NIHSS) inter-group comparison. Cortico-subcortical lesioned patients (CS), Subcortical lesioned patients (S).

Mean results of neurological evaluations are presented in Figure 1. No significant differences between CS and S were found.

### Frequency-Specific Power Measures

We found significantly higher Delta power in both groups of stroke patients compared to healthy subjects. Median and IQR values for band powers are presented in Table 2.

**Table 2:** EEG power spectral density in Delta and Alpha band, Delta/Alpha Ratio (DAR) and pairwise derived Brain Symmetry Index (pdBSI) synthetic indices on the whole scalp for Healthy, CS, S patiens.

	Healthy	CS	S	Mann Whitney value Healthy-CS	Mann Whitney value Healthy-S	Mann Whitney value CS-S
**Delta**	14.83 (13.17)	29.69 (19.02)	24.17 (14.81)	−2.98**	−2.64**	−1.19
**Alpha**	4.89 (13.43)	6.26 (4.69)	8.22 (20.47)	−0.18	−1.68	−2.18*
**DAR**	2.02 (4.07)	5.16 (3.86)	2.49 (3.97)	−2.21*	−0.76	−2.07*
**pdBSI**	0.2 (0.06)	0.22 (0.07)	0.28 (0.11)	−1.67	−2.81**	−2.11*

Median and Interquartile Range (IQR) values are described. Mann Whitney coefficient between groups. *p < 0.05; **p < 0.01.

### Brain Activity Group Comparison

**Figure 2:**
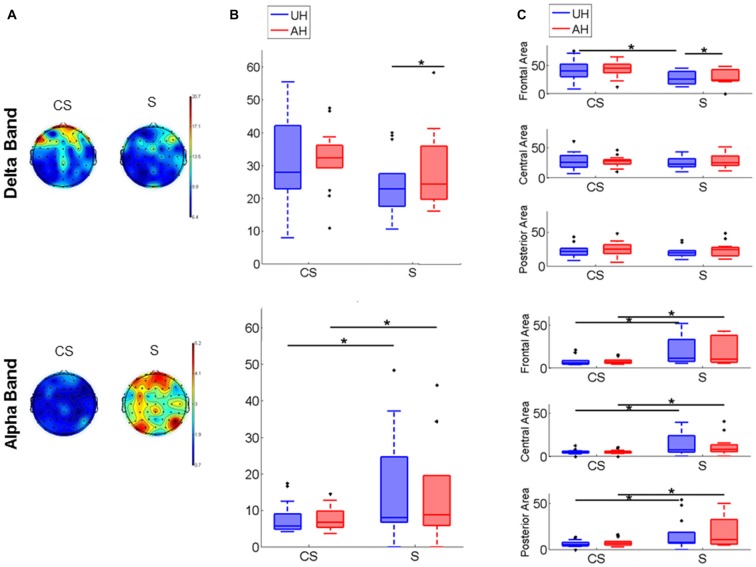
Power spectrum. **(A)** Power spectrum density (PSD) maps of Delta and Alpha EEG frequency band. Figure shows the Cortico-subcortical stroke patients (CS) scalp distribution on the left and the Subcortical stroke patients (S) scalp distribution on the right. To represent the average map of each group all stroke lesions were considered as affecting the right hemisphere. **(B)** Power spectral density box-plot of Delta and Alpha bands, Median and Interquartile Range (IQR) of power of unaffected hemisphere (UH) and affected hemisphere (AH) for Cortico-subcortical lesion (CS) and Subcortical lesion (S). **(C)** PSD box-plot of Delta and Alpha EEG frequency band, Median and IQR of power of Frontal Area, Central Area and Posterior Area for Cortico-subcortical lesion (CS) and Subcortical lesion (S). *p < 0.05.

PSD map of Delta and Alpha band distribution in CS and S is depicted in Figure 2A for a visual representation; to represent the average map of each group all stroke lesions were considered as affecting the right hemisphere. Inter-group comparison showed that Alpha band power was significantly higher in S than in CS in the whole scalp (*z* = −2.18, *p* < 0.05; Table 2), in each hemisphere (UH: *z* = −2.14, *p* < 0.05; AH: *z* = −1.96, *p* < 0.05; Figure 2B) and in each area (UH: frontal *z* = −2.39, *p* < 0.05, central *z* = −2.74, *p* < 0.05, posterior *z* = −2.07, *p* < 0.05; AH: frontal *z* = −2.02, *p* < 0.05, central *z* = −2.79, *p* < 0.05, posterior *z* = −1.95, *p* < 0.05; Figure 2C). Delta band power was significantly higher in CS than in S in the frontal area of the UH (*z* = −2.37, *p* < 0.05; Figure 2C). Intra-group comparison showed that Delta activity was significantly different between the AH and the UH in S patients (Delta AH > Delta UH) both in the whole hemisphere (*z* = −1.99, *p* < 0.05, Figure 2B) and in frontal area (*z* = −2.21, *p* < 0.05, Figure 2C), while no significant differences between the two hemispheres were found either in CS nor in Healthy subjects.

### Quantitative Indices

Median values for quantitative indices have been presented in Table 2.

**Figure 3:**
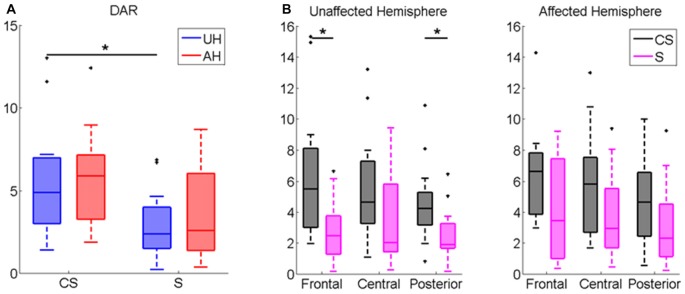
Delta/Alpha Ratio (DAR) Index computed for **(A)** UH and AH and for **(B)** Frontal, Central and Posterior Area in each hemisphere (UH and AH). Median and IQR of DAR in Cortico-subcortical lesioned patients (CS) and Subcortical lesioned patients (S). *p < 0.05.

DAR index was significantly higher in CS compared to healthy subjects (*z* = −2.21, *p* < 0.05). Patients Inter-group comparison showed that DAR was significantly higher in CS compared to S in the whole scalp (*z* = −2.07, *p* < 0.05, see Table 2), and more specifically in the UH (*z* = −2.48, *p* < 0.05, Figure 3A), particularly in frontal and posterior areas (*z* = −2.57, *p* < 0.05; *z* = −2.55 *p* < 0.05, respectively, Figure 3B). Intra-group comparison for DAR did not show any significant result.

**Figure 4:**
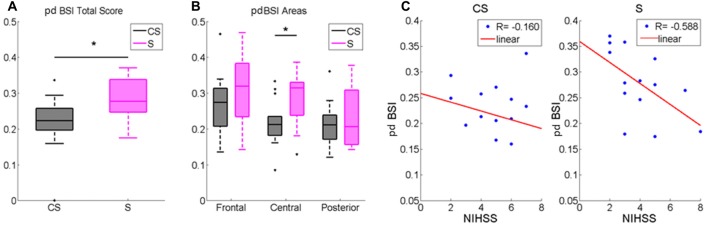
Pairwise derived brain symmetry index (pdBSI) computed for **(A)** the whole scalp and for **(B)** Frontal, Central and Posterior Areas. Median and IQR of pdBSI in Cortico-subcortical lesioned patients (CS) and Subcortical lesioned patients (S). *p < 0.05. **(C)** pdBSI and NIHSS score correlation in Cortico-subcortical lesion group (CS) and in Subcortical lesion group (S).

pdBSI was significantly higher in S compared to healthy subjects (*z* = −2.81, *p* < 0.01). Patients inter-group comparison showed a higher brain asymmetry in S patients both in the whole scalp (*z* = −2.11; *p* < 0.05, Table 2, Figure 4A) and in central area (*z* = −2.34; *p* < 0.05, Figure 4B).

### Spearman Correlations

Time since stroke did not correlate with EEG parameters (Delta and Alpha bands, pdBSI and DAR) in both groups.

We found that pdBSI was negatively correlated with NIHSS in S (*R* = −0.588, *p* < 0.05): a higher asymmetry matched a better clinical status (Figure 4C).

## Discussion

In this study we aimed to characterize brain electrical activity through qEEG analysis in a cohort of stroke patients recruited in subacute phase and subdivided on the basis of lesion location (cortico-subcortical and subcortical lesions). We found, in agreement with literature, a different bilateral cortical activity in stroke patients, with specific characterization related to lesion location (Luft et al., 2004; Niedermeyer, 2005; Grefkes and Ward, 2014).

Previous fMRI studies described activation patterns in stroke patients with a different lesion location. In detail whereas standard motor circuitry is involved in subcortical stroke, alternative networks are recruited after cortical stroke patients (Luft et al., 2004). Besides these relevant fMRI results, it is well known that brain functions may be associated to specific EEG band power activity (Kaplan and Rossetti, 2011; Finnigan and van Putten, 2013; Rossiter et al., 2014), even if up to now few information are available about brain activity characterization based on stroke location using qEEG.

In our study we found a different distribution of the low waves activity in the two groups. Delta activity was characterized by an asymmetric distribution in subcortical patients and symmetrical distribution in cortico-subcortical patients. In subcortical patients the interhemispheric imbalance was related to the degree of clinical impairment.

The occurrence of Delta oscillation particularly at electrodes overlying the ischemic region is the most common outcome. Delta activity is believed to originate in neurons in the thalamus and in deep cortical layers, and it may reflect hyperpolarization and inhibition of cortical neurons, resulting in deafferentation of neural activity (John and Prichep, 2006). Abnormal Delta increases are often associated with the primary injury location (neural tissue, functionally affected but without necrosis) and deafferented regions (Leon-Carrion et al., 2009; Kaplan and Rossetti, 2011). Our study showed a higher Delta-band power in the AH compared to the unaffected one in subcortical patients. The asymmetric distribution of Delta waves in subcortical stroke patients contributed to a greater interhemispheric power asymmetry, also described with the higher pdBSI value compared with CS. This parameter was already considered as an index of infarct volume in the first hours after stroke (Sheorajpanday et al., 2009), even if a specific behavior of pdBSI in relation to the level of the ischemic area has not been described before. Moreover, the fact that different pdBSI values between the two groups were found specifically in central electrodes indicates a specific involvement of sensorimotor areas in the post-lesional rearrangement. Furthermore, our results showed a negative correlation between pdBSI and NIHSS score in S patients, i.e., the greater the electrical asymmetry, the better the clinical status. The present study importantly described that the pdBSI index could be explicative of neurological status only in subcortical stroke and highlighted the direct relation between brain post lesional reorganization and functional status in this type of lesions. This result is in agreement with previous studies, which described the relation between subcortical lesion and clinical status, with a specific involvement of UH activity (Thickbroom et al., 2015; Lamola et al., 2016). One of these studies showed a direct correlation between the UH excitability and the clinical scales in the subacute stroke phase (Thickbroom et al., 2015). Moreover, the reduction of unaffected intracortical inhibition in subcortical stroke patients played an important role in effective motor recovery at 3 months after the acute event (Lamola et al., 2016). While these studies described the role of motor cortex and cortico-spinal tract in motor function, the innovative result of our study is the description of the specific bilateral process in subcortical stroke involved in the patients’ functional status by means of electrical cortical activity analysis. In this regard, more than previous studies, the EEG indices could highlight the implication of the UH functionality in patients’ clinical status.

CS patients were characterized by bilateral Delta-band activity distribution. The dominance of Delta waves was associated with wider brain lesion in several studies (Kotchoubey et al., 2005; Leon-Carrion et al., 2008, 2009). A previous study described an increase of contralesional Delta power in Middle Cerebral Artery ischemic stroke patients studied in the acute phase (within 10 days after stroke; Assenza et al., 2013), whereas in our data this mechanism persisted in subacute phase (from 10 days to 45 days after stroke). This phenomenon can be explained by an altered functionality in one area distant from the affected one, but functionally connected with it, called “diaschisis”, that can be mediated by hemodynamic or electrophysiological impairments (Assenza et al., 2013). A recent review accurately described this mechanism as “transhemispheric diaschisis”, which represents a decrease in metabolism in the contralateral cortex by interruption of transhemispheric pathways (Carrera and Tononi, 2014).

The different representation of Delta activity in the two groups is also confirmed by DAR values, which were significantly higher in CS compared to S, both in the whole scalp and in the UH. Finnigan et al. (2016) found that DAR is the most accurate index for discriminating between radiologically-confirmed acute ischemic stroke and age-matched controls, identifying 3.7 as the optimal DAR threshold value. Interestingly, we found supra-threshold values in CS patients and below-threshold values in S patients. This datum indicates that the DAR is a valuable index to discriminate the anatomical level of the lesion. Moreover previous studies showed a predictive function of DAR on motor recovery in acute stroke patients (Finnigan and van Putten, 2013; Schleiger et al., 2014). In detail the authors described a significant relation between DAR assessed in the first 48 h after stroke and NIHSS assessed at the same timing and after 1 month. Our study investigated the relation between brain activity and functional status in subacute phase but we did not find significant results. Presumably the different outcome is due to the timing of evaluation: in already cited literature patients were assessed in the early acute phase, while our data were collected between 10 days and 45 days after the acute event and this could modify the explicative role of DAR. Further longitudinal studies are needed in order to verify the predictive role of DAR in the subacute phase.

Another interesting result is that subcortical patients showed a higher Alpha power activity in both hemispheres. The understanding of the physiological nature of Alpha rhythm is still open to debate (Gonçalves et al., 2006). Although the generation of Alpha is not yet fully understood, animal and human research points out that the thalamus interacts with the cortex and the nucleus reticularis to oscillate synchronously in the Alpha frequency range (John and Prichep, 2006). Some studies refer to Alpha activity as a central timing mechanism regulating cortical afferent and efferent signals with spread rhythm within the cerebral cortex (Lopes da Silva et al., 1980; Manshanden et al., 2002). In other studies Alpha activity was related to attention mechanisms, including maintaining an optimal cerebral arousal state (Goldman et al., 2002; Gómez et al., 2006). In relation to the prognosis after brain injury, several studies have reported that preservation of Alpha activity is indicative of neuronal survival in ischemic regions and of a good prognosis (Juhász et al., 1997; Niedermeyer, 2005; Leon-Carrion et al., 2009). Moreover an old study conducted on stroke patients in acute phase described a bilateral preservation of Alpha activity suggesting a higher probability of circumscribed subcortical infarct (Juhász et al., 1997). Accordingly, our results showed a higher bilateral Alpha activity in subcortical group of post stroke patients compared to cortico-subcortical lesioned patients.

This study has a few limitations. First, the sample size was relatively small (*n* = 30) and results have to be confirmed in further studies with a greater sample. Second, the disease stage varied from 10 days to 45 days after the acute ischemic event, so the results could be affected by the width of the time window.

## Conclusion

Our results pointed out the characterization of brain activity between subcortical and cortico-subcortical subacute stroke patients. Subcortical stroke patients showed a higher Alpha power activity and an asymmetric distribution of Delta activity. In this kind of lesions the interhemispheric imbalance was related to the degree of clinical status. Differently, cortical involvement determined a scattered increase of low-frequency Delta activity in both hemispheres. This study explored electrical brain activity differences between stroke patients with cortico-subcortical and subcortical lesion location with the selection of distinct quantitative indices (pdBSI and DAR). However, longitudinal studies are needed to correlate neurophysiological data to the progression of recovery. This will allow a more precise definition of diagnostic-therapeutic algorithms in stroke patients.

## Author Contributions

CF recruited patients, performed neurophysiological evaluations, executed data analysis, interpreted the results and drafted the manuscript; FB performed clinical and neurophysiological evaluations, helped to interpret the results and to draft the manuscript; GL performed clinical and neurophysiological evaluations, helped to interpret the results and revised the manuscript; AP helped perform data analysis and revised the manuscript; FA performed EEG signal preprocessing and revised the manuscript; SM was responsible for data analysis and statistics, supervised the experiment; BR supervised the manuscript; CC designed the study, was responsible for patients’ enrollment, supervised the experiment, contributed to interpretation of data and revised manuscript. All authors read and approved the final manuscript.

## Conflict of Interest Statement

The authors declare that the research was conducted in the absence of any commercial or financial relationships that could be construed as a potential conflict of interest.
